# Toxicity and Human Health Assessment of Air Pollutants

**DOI:** 10.3390/toxics13100884

**Published:** 2025-10-16

**Authors:** Ting Wang, Tingting Ku, Jia Xu

**Affiliations:** 1 Tianjin Key Laboratory of Urban Transport Emission Research, College of Environmental Science and Engineering, Nankai University, Tianjin 300071, China; 2 Shanxi Key Laboratory of Coal-based Emerging Pollutant Identification and Risk Control, Research Center of Environment and Health, College of Environment and Resource Sciences, Shanxi University, Taiyuan 030006, China; kutingting@sxu.edu.cn; 3 State Key Laboratory of Environmental Criteria and Risk Assessment, Chinese Research Academy of Environmental Sciences, Beijing 100012, China; xu.jia@craes.org.cn

## 1. Introduction

Air pollution has been shown to be responsible for the morbidity and mortality of a variety of major diseases [1,2,3]. The World Health Organization (WHO) estimates that almost all of the global population breathes air that exceeds the WHO’s guidelines and contains high levels of pollutants [4,5]. To date, however, the key constituents of air pollution that impact health and the potential mechanisms remain largely unstudied [6]. In addition, comprehensive assessments of evidence from in vitro and in vivo studies, high-quality cohort studies, and high-resolution model simulations of health impacts are still substantially sparse [7].

This Special Issue, entitled “Toxicity and Human Health Assessment of Air Pollutants”, aims to understand the mechanisms of the health effects caused by air pollution and ensure accurate exposure assessments around ambient environments and inside human bodies. The collated articles employ a range of approaches—from epidemiological cohorts and toxicological studies—to elucidate the linkage between air pollutant exposure and the mechanisms related to health effects. The evidence presented in this Special Issue provides valuable insights for controlling air pollution and reducing the burden on public health.

## 2. An Overview of Published Articles

The eleven articles published in this Special Issue collectively advance our understanding of the health impacts of air pollution exposures, which can be categorized into four main research directions: elucidating underlying toxicity mechanisms, identifying critical exposure windows and vulnerable populations in epidemiological studies, exploring the impact on postoperative populations, and estimating the association between air pollutant exposure and health effects.

Firstly, several studies delve into the molecular mechanisms that may mediate the link between air pollution exposures and adverse health outcomes. Wang et al. (Contribution 1) indicated that PM_2.5_ induced glomerular hyperfiltration in female mice by affecting RAS/KKS imbalances and the regulation of TGF. They innovatively unveiled the association between subchronic exposure to PM_2.5_ and early kidney injury, highlighting its gender dependence. Gao et al. (Contribution 2) revealed that diesel exhaust caused disrupted lipid metabolism, acute liver injury, and more severe inhibition of cell proliferation and oxidative damage compared to gasoline exhaust particles. Fu et al. (Contribution 3) emphasized that cooking oil fumes (COFs), fine particulate matter (PM_2.5_), and cigarette smoke (CS) could decrease the viability of epidermal HaCaT cells and dermal fibroblast (FB) cells, promote the secretion of pro-inflammatory cytokines IL-1α and TNF-α in HaCaT cells, increase intracellular ROS levels, and downregulate the mRNA expression of collagen I, III, IV, and VII in FB cells, thus leading to skin damage. Yan et al. (Contribution 4) summarized the molecular mechanisms involved in the health effects of PM_2.5_ on adverse birth outcomes regarding cardiopulmonary and neurological developments, primarily including transcriptional and translational regulation, oxidative stress, inflammatory response, and epigenetic modulation.

Secondly, three papers assess the health impact of air pollution exposure by human epidemiology, highlighting critical susceptibility windows, vulnerable subpopulations, and the interactions of mixed exposures. Chen et al. (Contribution 5) found that prenatal exposure to incense-burning smoke (IBS) increased the risk of the presence of obesity in preschoolers, prenatal exposure to IBS combined with a lower frequency of early outdoor activity or a shorter duration of outdoor activity from the ages of 1 to 3 years increased the risk of obesity in preschoolers, and there were additive interactions between prenatal exposure to IBS and postnatal outdoor activity on obesity. Yang et al. (Contribution 6) employed a two-sample Mendelian randomization (MR) analysis that provided genetic evidence for a null causal relationship between air pollutants and NAFLD in the European population. The associations observed in epidemiological studies could be partly attributed to confounders. Han et al. (Contribution 7) illustrated that O_3_ exposure was negatively associated with vision disorder. In addition, subgroup analyses revealed that PM_2.5_ exposure was significantly correlated with the risk of glaucoma and age-related macular degeneration and that children and adolescents were more susceptible to NO_2_ and PM_2.5_ than adults.

Thirdly, two papers evaluate the impact of air pollutant exposure in postoperative populations. Urbanowicz et al. (Contribution 8) suggested that chronic exposure to ambient air pollutants such as PM_2.5_ may be regarded as an additional risk factor in patients after surgical revascularization with left ventricular dysfunction. Zhang et al. (Contribution 9) found that the presence of the heavy metal of cadmium (Cd) in an atmospheric milieu acts as a catalyst in the progression of tumorigenesis within murine models of colon cancer (CC). The implementation of intestinal stents demonstrates a mitigating effect on tumor incidence within these CC murine models. The effect of Cd on the invasive effect of intestinal stents in the cancerous colon is not significant.

Finally, two papers assess the health effects of air pollutant exposure by modeling approaches. Wang et al. (Contribution 10) used the VSL (value of statistical life) method to find that the number of deaths attributed to PM_2.5_ in Beijing in 2021 fell by 33.74 percent from 2016, while health economic losses will increase by USD 4.4 billion as per capita disposable income increases annually. Wu et al. (Contribution 11) developed spatial models for NO_2_ and PM_2.5_ and conducted exposure assessment in Beijing, China. The results showed that the partial least squares (PLS)–ordinary kriging (OK) models for NO_2_ and PM_2.5_ had the best performance compared to other spatial modeling algorithms. Hence, the exposure misclassification made by choosing different modeling approaches should be carefully considered, and the resulting bias needs to be evaluated in epidemiological studies.

## 3. Conclusions

The studies in this Special Issue provide a new understanding of the health impacts of air pollution. They elucidate toxicity mechanisms, identify critical windows and vulnerable populations in epidemiological studies, assess effects on postoperative patients, and estimate health impacts via modeling approaches.

The molecular mechanisms linking air pollution were related to kidney injury, liver damage, and skin aging through oxidative stress, inflammation, and epigenetic changes. Epidemiological research identified early life as a critical exposure window, with air pollution associated with childhood obesity and vision disorders. Postoperative studies indicate that chronic PM_2.5_ exposure worsened outcomes in patients with heart failure, and heavy metals like cadmium may accelerate tumor progression. Epidemiological studies show that despite reduced PM_2.5_-attributable deaths in Beijing, economic costs have risen, emphasizing the necessity for careful exposure assessment in future studies.

This Special Issue highlights the multifaceted health threats of air pollution while underscoring methodological considerations in exposure assessment and the importance of protecting vulnerable subgroups.

## Data Availability

The data presented in this manuscript will be made available by the authors upon request.
